# Socioeconomic and demographic predictors of selected cardiovascular risk factors among adults living in Pohnpei, Federated States of Micronesia

**DOI:** 10.1186/1471-2458-14-895

**Published:** 2014-08-31

**Authors:** Gwendolyn M Hosey, Marcus Samo, Edward W Gregg, Diane Padden, Sandra Garmon Bibb

**Affiliations:** Division of Diabetes Translation, Centers for Disease Control and Prevention, National Center for Chronic Disease and Health Promotion, Mailstop K10, 2877 Brandywine Rd, Atlanta, GA 30341 USA; Department of Health and Social Affairs, Federated States of Micronesia, P.O. Box PS 70, Palikir, Pohnpei, 96941 Federated States of Micronesia; Uniformed Services University, Graduate School of Nursing, 4301 Jones Bridge Road, Bethesda, Maryland 20814 USA

**Keywords:** Micronesia, Health disparities, Chronic disease, Cardiovascular disease risk factors, Health determinants

## Abstract

**Background:**

The burden of cardiovascular disease (CVD) is increasing in low-to-middle income countries (LMIC). Although strong evidence for inverse associations between socioeconomic position and health outcomes in high-income countries exists, less is known about LMIC. Understanding country-level differences is critical to tailoring effective population health policy and interventions. We examined the association of socioeconomic position and demographic characteristics in determining CVD risk factors among adults living in Pohnpei, Federated States of Micronesia.

**Methods:**

We used data from the cross-sectional World Health Organization’s STEPwise approach to surveillance 2002 Pohnpei dataset and logistic regression analyses to examine the association of socioeconomic position (education, income, employment) and demographics (age, sex) with selected behavioral and anthropometric CVD risk factors. The study sample consisted of 1638 adults (642 men, 996 women; 25–64 years).

**Results:**

In general, we found that higher education (≥13 years) was associated with lower odds for daily tobacco use (odds ratio [OR]: 0.46, confidence interval [CI]: 0.29–0.75, p = 0.004) and low physical activity (OR: 0.55, CI: 0.34–0.87, p = 0.027). Men had over three times the odds of daily tobacco use than women (OR: 3.18, CI: 2.29–4.43, p < 0.001). Among women, paid employment nearly doubled the odds of daily tobacco use (OR: 1.72, CI: 1.08–2.73, p = 0.006) than unemployment. For all participants, income > $10,000 was associated with over twice the odds of high blood pressure (BP) (OR: 2.24, CI: 1.43–3.51, p = 0.003), versus lower-income (<$5,000). Men had over twice the odds of high BP (OR: 2.01, CI: 1.43–2.83, p < 0.001) than women. Paid employment nearly doubled the odds of central obesity with the magnitude of association increasing by more than 20% adjusted for sex and age. Men reporting paid employment had three times the odds of central obesity (OR: 3.00, CI: 1.56–5.78, p < 0.001) than those unemployed.

**Conclusion:**

Our analysis revealed associations between socioeconomic position and selected CVD risk factors, which varied by risk-factor, sex and age characteristics, and direction of association. The 2002 Pohnpei dataset provides country-level baseline information; further population health surveillance might define trends. Stronger country-level data might help decision-makers tailor population-based prevention strategies.

**Electronic supplementary material:**

The online version of this article (doi:10.1186/1471-2458-14-895) contains supplementary material, which is available to authorized users.

## Background

Globally, more than 80% of cardiovascular disease (CVD) deaths occur in low- to middle-income countries (LMIC) [1, 2]. Moreover, in LMIC, 29% of deaths from chronic noncommunicable diseases occur before the age of 60 years, compared with 13% in high-income countries [1]. By 2030, three-quarters of all deaths worldwide (estimated) will be related to chronic noncommunicable diseases, exceeding the number of deaths from infectious diseases (including HIV/AIDS, tuberculosis, and malaria), maternal and perinatal health complications, and nutritional disorders combined [3].

A complex interplay of sociodemographic trends, including population aging and economic change, explain the epidemiological transition from a burden of morbidity/mortality dominated by infectious disease to chronic disease [4]. Compared with the circumstances in high-income countries, in which burden has increased over the course of a century, the increase within LMIC is occurring more rapidly [5, 6]. Between 1990 and 2020, the age-specific ischemic heart disease mortality rate is projected to increase by 120% for women and 137% for men in LMIC, compared with increases of 30% for women and 60% for men in high-income countries (largely attributable to population growth of adults aged ≥65 years) [7].

For most high-income countries, recent decades have seen a decline in CVD mortality partly due to improved treatment and care, primary prevention, and declines in risk factors such as smoking [8, 9]. In LMIC, upward population-level trends in higher risk lifestyle behaviors (e.g., tobacco use, physical inactivity, and inadequate fruit and vegetable consumption) and the increasing prevalence of underlying metabolic risk factors (e.g., obesity, high blood pressure, dyslipidemia, and diabetes) are associated with increased burden of CVD [10, 11]. For example, mortality projections for 2030 indicate that seven in ten tobacco-related deaths worldwide would occur within LMIC [12]. Additionally, between 1998 and 2008, the largest increase in mean body mass index (BMI) worldwide rose 0.4 kg/m^2^ in men and 0.5 kg/m^2^ in women per decade (aged ≥20 years) with the largest rise in BMI (2.0 kg/m^2^ per decade) occurring in nine countries of Oceania (Australia, New Zealand, and Pacific Island Nations) [13].

**Figure 1:**
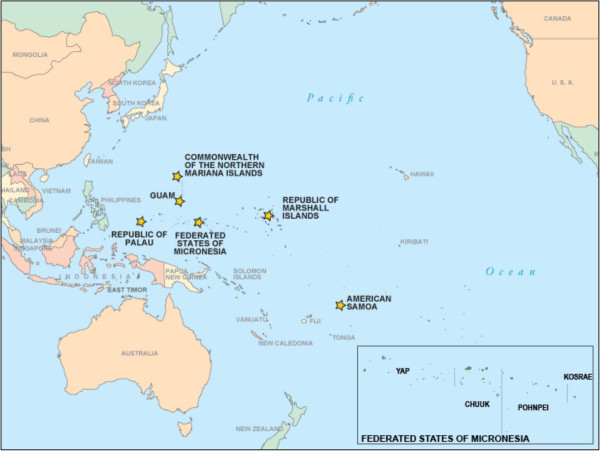
**Map of the US Associated Pacific Island Jurisdictions, Federated States of Micronesia.**

Since 2002, the Federated States of Micronesia (FSM) has been developing capacity for chronic disease surveillance; resultant data are available that may provide an opportunity for extending the understanding of country-level associations between socioeconomic position and CVD risk factors within LMIC. The World Bank classifies FSM as a LMIC with a gross domestic product income (GDP) at purchasing power parity of $3,165 per capita (2012 estimate [est.]), compared with $50,700 GDP per capita in the United States (2012 est.) [14]. FSM, a constitutional federation, with a Compact of Free Association with the United States, is composed of four island states: Chuuk, Kosrae, Pohnpei, and Yap (Figure 1).

Research evidence, primarily from high-income countries, shows inverse relationships between socioeconomic position and CVD risk factors [15–17]. Most studies conducted within LMIC have focused on geographic (i.e., north-south) and between-country differences, rather than on gaps between social groups within countries [18]. Understanding the country-level differences in associations between socioeconomic position and chronic noncommunicable disease is critical to tailoring effective population health policy and interventions aimed toward decreasing CVD risk factors within LMIC [19, 20]. Our research helps to fill that gap by describing the association between socioeconomic position and CVD risk factors in a population-based sample of the adult population living in Pohnpei, FSM.

## Methods

### Design

We performed a descriptive cross-sectional secondary analysis of data from the 2002 STEPwise approach to surveillance (STEPS) in Pohnpei [21]. Detailed STEPS design and methodology are available at: http://www.who.int/chp/steps/en/. The STEPS dataset, developed and administered by the World Health Organization (WHO), includes a sequential three-step process to collect information on risk factors for chronic diseases: 1) a personal interview, 2) anthropometric measurements, and 3) a biochemical assessment.

Prior to accessing the dataset, we completed an assessment of it for completeness and quality (Additional file 1) using established secondary analysis assessment criteria [22–24] and publicly available STEPS metadata archives. Generally, the assessment showed that the dataset was appropriate for secondary analysis with adequate power, a representative sample, validated instrumentation, standardized data collection, and limited issues regarding missing data. Thus, we were able to use the dataset to provide reliable data necessary for the primary objective of this study.

### Created sample and sample size considerations

The 2002 STEPS sampling process used a multistage, probabilistic, cluster design (based on 2000 Pohnpei census enumeration areas) to randomly select households for participation. Data were obtained from 1638 adults (78% response rate) aged 25–64 years. Sample size calculations and details related to sampling procedures used in collecting STEPS data for the primary study are reported elsewhere [21]. We included the entire 2002 STEPS Pohnpei dataset to ensure that the study sample was representative of the overall population.

### Ethical considerations

Before we downloaded data, the Uniformed Services University Institutional Review Board and the FSM Department of Health and Social Affairs approved this study.

### Study variables

Table 1 provides a summary of conceptual definitions and measures for the variables used in our study analyses. A brief description of outcome and predictor variables is provided below.

**Table 1:** **Summary of variable names, conceptual definition, and measures using the 2002 STEPS Pohnpei, FSM data set**

Variable	Conceptual definition	Measures ^a^
**Socioeconomic position**	Associated with an individual’s relative position within a social structure	Self-report of educational attainment, annual household income, occupation/employment
**Demographic characteristics**	Information concerning an individual’s age and sex	Age in years and sex
**Behavioral characteristics**	Information concerning an individual’s lifestyle behaviors associated with CVD risk (i.e., stroke, myocardial infarction, coronary artery or peripheral vascular disease)	Self-report of tobacco use (daily use smoke or smokeless), physical activity (moderate level of physical activity [work, travel, leisure] on 5 or more days/ week)
**Anthropometric characteristics**	Information concerning an individual’s anthropometric features associated with CVD risk	Waist circumference (excluding pregnant women); mean systolic and diastolic blood pressure (calculated using last two of three BP)

*Abbreviations*: STEPS World Health Association STEPwise approach to surveillance, *FSM* Federated States of Micronesia, *CVD* Cardiovascular risk, *BP* Blood pressure.

^a^Detailed descriptions for measures used in this study available at World Health Association STEPwise approach to surveillance (STEPS) available at: http://www.who.int/chp/steps/en/.

### Outcome variables

#### Cardiovascular risk factors

Data on four recognized CVD risk factors were used in our analysis: 1) daily tobacco use, 2) low physical activity, 3) high blood pressure (BP), and 4) central obesity (Table 1). Participants reporting daily tobacco use and a less-than-moderate level of physical activity (i.e., less than 30 minutes of activity, five or more days a week) were defined as being at risk [16]. We used the mean of the two most recent BP measurements to create systolic and diastolic BP variables. We considered participants to have high BP if they had a systolic value ≥140 mmHg or a diastolic value ≥90 mmHg; or if they reported a diagnosis of hypertension or if they reported using BP medication within the last year [25]. We defined central obesity as waist circumference >102 centimeters (cm) in men and >88 cm in women. These cut-off points for central obesity are associated with a substantially increased risk for one of three indicators of CVD risk: high blood pressure, diabetes, or hyperlipidemia [26].

### Predictor variables

#### Socioeconomic position

We used self-reported educational attainment, estimated annual household income, and employment status as indicators of socioeconomic position. In addition to other benefits, income and employment status can also provide access to resources to promote health [15].

### Education

Educational attainment is a preferred socioeconomic position indicator, as education can be determined for all individuals and is fairly stable after early adulthood. Additionally, education is a significant determinant of a person’s economic potential and acquisition of life-skills relative to adopting health-promoting behaviors [15]. Using self-report of years of education completed, we categorized participants into one of three educational-level groups: primary (<9 years), secondary (9–12 years), and postsecondary (≥13 years). Persons with a primary education were used as the reference group in the analysis.

### Income

We included estimated annual household income in the analysis, categorizing participants into one of three groups: low (<$5,000), middle ($5,000–$10,000), or high income (>$10,000). For the analysis, persons in a household with low income < $5,000 were used as the reference group. An “unknown income” category was included to account for missing values for the income field in the primary dataset [27].

### Employment status

For employment status, we categorized participants into one of three groups: paid, unpaid (i.e., retiree, volunteer, student, homemaker), and unemployed. For the analysis, persons who were unemployed were used as the reference group.

### Covariates

We included sex and age in the analysis as covariates. The literature supports sex and age possibly being mediating or confounding factors in the association between socioeconomic position and CVD risk factors. Age and sex may affect risk factor prevalence through variations in both biological characteristics and societal influences that vary across societies [15, 28].

### Statistical methods

Data were analyzed using SPSS version 20.0 complex samples module that accounts for the complex sampling design used in the STEPS survey, correctly calculating standard errors with weighted data. We applied sex-age structure survey weights (standardized to the FSM 2000 census for Pohnpei) to provide results representative of the adult Pohnpeian population aged 25–64 years. After data cleaning and recoding, we completed descriptive statistics for all study variables. We also completed exploratory analyses, using chi-square and one-way analysis of variance (with tests of normality and other assumptions and post-hoc comparisons) reported elsewhere [29]. Through this process, we identified the associations of socioeconomic position and demographic characteristics with outcome variables (using *a priori* probability value of <0.25, or had clinical relevance) for inclusion in the multivariate logistic regression models that this paper addresses.

We used multivariate logistic regression models to determine the extent to which socioeconomic position predicted CVD risk factors. First, we examined the crude associations between primary socioeconomic predictors (i.e., education, income, and employment) and each outcome measure. We also examined all three predictors and sex and age included as covariates. *A priori,* we also developed strata models based on evidence that sex and age may be confounding factors in the association between socioeconomic position and CVD risk factors [15, 28]. Magnitude of effect was calculated using the simple difference between crude and adjusted odds ratio estimates with values greater than 10% considered meaningful. Results were considered significant if p was less than 0.05 (p < 0.05).

## Results

We provide selected characteristics of the sample dataset, collectively and stratified by sex, for the overall population (Table 2). Most respondents reported a primary-level education (<9 years) and incomes at < $5,000. More than one-half of the participants reported paid employment. Over one-third of male respondents reported daily tobacco use, and over one-third of female respondents had central obesity.

**Table 2:** **Selected characteristics of sample by sex and collectively, using the 2002 STEPS Pohnpei, FSM data set** ^**a**^

Characteristic (measure)	Male n (%)	Female n (%)	Total sample n (%)
N	642	996	1638
**Age range**			
25–34 y	176 (36.8)	321 (39.2)	496 (38.0)
35–44 y	187 (32.5)	313 (31.2)	500 (31.9)
45–54 y	184 (21.1)	247 (19.6)	431 (20.4)
55–64 y	95 (09.5)	115 (10.0)	210 (09.8)
**Education (highest level completed)**			
Primary (<9 y)	315 (52.2)	568 (59.1)	883 (55.6)
Secondary (9–12 y)	183 (31.4)	302 (34.1)	485 (32.7)
Post-secondary (≥13 y)	99 (16.4)	57 (06.8)	156 (11.6)
**Income (estimated annual household)**			
Low (< $5,000)	298 (47.5)	486 (49.3)	784 (48.4)
Middle ($ 5,000–$10,000)	121 (18.4)	170 (16.8)	291 (17.6)
High (> $10,000)	65 (09.1)	93 (09.1)	158 (09.1)
Unknown	158 (25.0)	247 (24.7)	405 (24.9)
**Employment** ^**b**^			
Paid employment	409 (67.7)	324 (36.1)	733 (52.3)
Unpaid	66 (09.8)	267 (29.2)	333 (19.3)
Unemployed	125 (22.5)	313 (34.6)	438 (28.4)
**Behavioral** ^**c**^			
Daily tobacco use	235 (37.7)	169 (16.7)	404 (27.3)
Low physical activity (<30 min/d, 5d/wk)	407 (72.6)	730 (88.6)	1137 (80.4)
**Anthropometric**			
Central obesity (waist circumference: M > 102 cm; F > 88 cm)	171 (21.3)	255 (33.9)	426 (27.4)
High BP (≥140/90 mmHG, use of BP medication, or self-report of hypertension diagnosis within last year)	209 (29.3)	209 (19.2)	418 (24.3)

*Abbreviations*: *FSM* Federated States of Micronesia, *N* Number, *y* Years, *d* Day, *wk* Week, *min* Minutes, *M* Male, *F* Female, *BP* Blood pressure, *cm* Centimeters.

^a^Using the 2002 STEPS Pohnpei, FSM data set; all estimates are sex–age standardized to the FSM 2000 Pohnpei Census; Percentages may not total 100 due to rounding. Behavioral variables are self-report; anthropometric are direct measures; central obesity excludes pregnant women.

^b^Unpaid category includes student, homemaker, volunteer, or retired.

^c^Daily tobacco use includes daily use of cigarettes, cigars, pipes, or smokeless tobacco; physically active defined using computed score: intensity (i.e., moderate or vigorous), duration, or metabolic rate.

We display the results from the logistic regression models (unadjusted and sex-age adjusted) predicting cardiovascular disease risk factors (Table 3). For daily tobacco use, the unadjusted model showed significant associations for each socioeconomic predictor; although except for educational attainment, the estimates for income and employment status were not significant in the sex-age adjusted model. Men had more than three times the odds of reporting daily tobacco use (sex-age adjustment; odds ratio [OR]: 3.18, 95%, confidence interval [CI]: 2.29–4.43, p < 0.001). Participants reporting a postsecondary educational level were significantly less likely to report daily tobacco use, with the magnitude of effect decreasing by 14.8% for sex-age adjustment.

**Table 3:** **Logistic regression analyses on association between socioeconomic demographic characteristics and cardiovascular risk factors, Pohnpei, FSM, 2002** ^**a**^

Predictor (measure)	Model 1 main effects		Final model main effects sex-age adjusted	
	OR (95% CI)	p	OR (95% CI)	p
**Daily tobacco use** ^**b**^				
**Education (highest level completed)**				
Primary (<9 y) (reference)	1		1	
Secondary (9–12 y)	0.71 (0.51–0.98)	0.022	0.68 (0.49–0.95)	0.004
Post–secondary (≥13 y)	0.54 (0.33–0.86)		0.46 (0.29–0.75)	
**Income (estimated annual household)**				
Low (<$5,000) (reference)	1		1	
Middle ($5,000–$10,000)	0.74 (0.47–1.17)	0.029	0.77 (0.50–1.20)	0.082
High (>$10,000)	0.46 (0.26–0.80)		0.50 (0.28–0.91)	
**Employment status** ^**c**^				
Unemployed (reference)	1		1	
Paid	1.76 (1.30–2.38)	< 0.001	1.33 (0.96–1.83)	0.201
Unpaid	0.90 (0.65–1.25)		1.10 (0.77–1.58)	
**Sex**				
Female	NA		1	
Male			3.18 (2.29–4.43)	< 0.001
**Age**				
<35 y (reference)	NA		1	
35–44 y			1.50 (1.13–1.99)	< 0.001
45–54 y			1.25 (0.92–1.70)	
>54 y			0.52 (0.30–0.91)	
**Low physical activity** ^**d**^				
**Education (highest level completed)**				
Primary (<9 y) (reference)	1		1	
Secondary (9–12 y)	0.91 (0.68–1.22)	0.002	0.92 (0.68–1.23)	0.027
Post–secondary (≥13 y)	0.46 (0.29–0.73)		0.55 (0.34–0.87)	
**Income (estimated annual household)**				
Low (<$5,000) (reference)	1		1	
Middle ($5,000–$10,000)	1.04 (0.67–1.61)	0.143	0.99 (0.62–1.60)	0.313
High (>$10,000)	1.63 (0.89–2.98)		1.47 (0.76–2.83)	
**Employment status** ^**c**^				
Unemployed (reference)	1		1	
Paid	1.08 (0.72–1.62)	0.045	1.35 (0.91–2.01)	0.158
Unpaid	1.94 (1.09–3.46)		1.60 (0.89–2.90)	
**Sex**				
Female	NA		1	
Male			0.33 (0.23–0.47)	< 0.001
**Age**				
<35 y (reference)	NA		1	
35–44 y			1.00 (0.65–1.52)	0.018
45–54 y			1.75 (1.20–2.55)	
>54 y			1.30 (0.77–2.18)	
**High BP** ^**e**^				
**Education (highest level completed)**				
Primary (<9 y) (reference)	1		1	
Secondary (9–12 y)	0.62 (0.41–0.93)	0.049	0.76 (0.49–1.17)	0.402
Post–secondary (≥13 y)	0.72 (0.42–1.21)		0.80 (0.45–1.39)	
**Income (estimated annual household)**				
Low (<$5,000) (reference)	1		1	
Middle ($5,000–$10,000)	1.19 (0.81–1.74)	< 0.001	1.06 (0.68–1.66)	0.003
High (>$10,000)	2.53 (1.74–3.68)		2.24 (1.43–3.51)	
**Employment status** ^**c**^				
Unemployed (reference)	1		1	
Paid	1.17 (0.94–1.45)	0.138	0.90 (0.69–1.17)	0.647
Unpaid	0.91 (0.59–1.42)		0.83 (0.52–1.32)	
**Sex**				
Female	NA		1	
Male			2.01 (1.43–2.83)	< 0.001
**Age**				
<35 y (reference)	NA		1	
35–44 y			1.94 (1.30–2.90)	< 0.001
45–54 y			3.77 (2.57–5.51)	
>54 y			6.08 (3.68–10.06)	
**Central obesity** ^**f**^				
**Education (highest level completed)**				
Primary (<9 y) (reference)	1		1	
Secondary (9–12 y)	1.12 (0.86–1.45)	0.179	1.14 (0.88–1.47)	0.315
Post–secondary (≥13 y)	0.78 (0.52–1.16)		0.85 (0.56–1.31)	
**Income (estimated annual household)**				
Low (<$5,000) (reference)	1		1	
Middle ($5,000–$10,000)	1.20 (0.82–1.75)	0.615	1.17 (0.79–1.72)	0.792
High (>$10,000)	1.28 (0.78–2.10)		1.21 (0.72–2.01)	
**Employment status** ^**c**^				
Unemployed (reference)	1		1	
Paid	1.57 (1.13–2.19)	0.001	1.90 (1.39–2.60)	< 0.001
Unpaid	1.60 (1.15–2.23)		1.47 (1.06–2.05)	
**Sex**				
Female	NA		1	
Male			0.50 (0.35–0.71)	< 0.001
**Age**				
<35 y (reference)	NA		1	
35–44 y			0.82 (0.57–1.18)	0.111
45–54 y			1.09 (0.77–1.54)	
>54 y			1.16 (0.68–1.98)	

*Abbreviations*: *FSM* Federated States of Micronesia, *BP* Blood pressure, *OR* Odds ratio, *CI* Confidence interval, *y* Year, *NA* Not applicable.

^a^Using the 2002 STEPS Pohnpei, FSM data set; all estimates are age–sex standardized to the FSM 2000 Pohnpei Census; p-values based on the Rao–Scott adjustment to χ^2^.

^b^Daily tobacco use includes daily use of cigarettes, cigars, pipes, or smokeless tobacco.

^c^Unpaid category includes student, homemaker, volunteer, or retired.

^d^Physically inactive includes <30 minutes/day of moderate activity on five or more days/week.

^e^High BP defined as BP ≥ 140/90 mmHG, use of BP medication, or self–report of hypertension diagnosis.

^f^Central obesity defined as waist circumference: Male > 102 cm; Female > 88 cm.

Significant inverse associations were found between low physical activity and postsecondary education, with the magnitude of effect weakened by 19.6% for sex-age adjustment. Compared with women, men had double the odds for high BP (OR: 2.01, CI: 1.43-2.83, p < 0.001). Participants reporting high income (>$10,000) had over twice the odds of high BP. Paid employment nearly doubled the odds of central obesity, with the magnitude of effect increasing by 21.0% for sex-age, compared with the unadjusted model (Table 3).

Table 4 shows results stratified by sex. For example, compared with unemployed women, women reporting paid employment had 1.72 times the odds (CI: 1.08–2.73, p = 0.006) of daily tobacco use and women reporting postsecondary education had significantly lower odds (OR: 0.17, CI: 0.04–0.76, p = 0.042) of daily tobacco use than those with a primary educational level. Men reporting a postsecondary educational level (OR: 0.50, CI: 0.28–0.88, p = 0.025) were significantly less likely to report low physical activity than those reporting a primary level. For men and women, odds of reporting high BP significantly increased by age group with over four times the odds for men aged 55–64 years as for men aged 25–34 years (OR: 4.58, CI: 2.41–8.71, p < 0.001) and women aged 55–64 years nearly 13 times the odds for high BP as those aged 25–34 years (OR: 12.98, CI: 5.29–31.86, p < 0.001). Women with high income had over twice the odds of high BP than those with low income (OR: 2.36, CI: 1.23–4.52, p < 0.001). Among men, paid employment was associated with a threefold increase in the odds for central obesity (OR: 3.00; CI: 1.56–5.78, p < 0.001).

**Table 4:** **Sex-stratified logistic regression models on association between socioeconomic demographic characteristics and cardiovascular risk factors, Pohnpei, FSM, 2002** ^a^

Predictor (measure)	Daily tobacco use ^b^		Physically inactive ^c^		High BP ^d^		Central obesity ^e^	
	OR (95% CI)	p	OR (95% CI)	p	OR (95% CI)	p	OR (95% CI)	p
**Male**								
**Education (highest level completed)**								
Primary (<9 y) (reference)	1		1		1		1	
Secondary (9–12 y)	0.70 (0.46–1.06)	0.062	1.01 (0.63–1.64)	0.025	0.51 (0.28–0.91)	0.062	1.02 (0.58–1.79)	0.659
Post–secondary (≥13 y)	0.54 (0.32–0.93)		0.50 (0.28–0.88)		0.72 (0.34–1.41)		0.78 (0.41–1.50)	
**Income (estimated annual household)**								
Low (<$5,000) (reference)	1		1		1		1	
Middle ($5,000–$10,000)	0.79 (0.42–1.51)	0.429	0.98 (0.54–1.79)	0.181	1.00 (0.59–1.71)	0.111	1.66 (0.83–3.35)	0.349
High (>$10,000)	0.53 (0.24–1.19)		1.95 (0.95–4.03)		2.00 (1.06–3.79)		1.59 (0.64–3.93)	
**Employment status** ^**b**^								
Unemployed (reference)	1		1		1		1	
Paid	1.26 (0.77–2.04)	0.140	1.43 (0.83–2.46)	0.280	1.10 (0.73–1.68)	0.403	3.00 (1.56–5.78)	<0.001
Unpaid	1.70 (0.98–2.95)		1.92 (0.76–4.83)		0.71 (0.33–1.55)		1.12 (0.47–2.63)	
**Age**								
25–34 y (reference)	1		1		1		1	
35–44 y	1.71 (1.08–2.70)	<0.001	1.01 (0.54–1.88)	0.005	1.57 (0.97–2.53)	<0.001	1.24 (0.60–2.57)	<0.001
45–54 y	1.17 (0.75–1.82)		2.14 (1.30–3.50)		2.93 (1.76–4.86)		3.36 (1.83–6.18)	
55–64 y	0.34 (0.15–0.78)		1.72 (0.90–3.26)		4.58 (2.41–8.71)		3.25 (1.57–6.73)	
**Female**								
**Education (highest level completed)**								
Primary (<9 y) (reference)	1		1		1		1	
Secondary (9–12 y)	0.69 (0.45–1.06)	0.042	0.68 (0.42–1.11)	0.258	1.41 (0.84–2.36)	0.365	0.96 (0.68–1.36)	0.428
Post–secondary (≥13 y)	0.17 (0.04–0.76)		0.65 (0.22–1.94)		1.12 (0.37–3.35)		0.68 (0.36–1.29)	
**Income (estimated annual household)**								
Low (<$5,000) reference	1		1		1		1	
Middle ($5,000–$10,000)	0.75 (0.39–1.44)	0.78	1.01 (0.49–2.08)	0.652	1.17 (0.59–2.29)	<0.001	0.92 (0.60–1.40)	0.979
High (>$10,000)	0.45 (0.22–0.96)		0.88 (0.39–1.97)		2.36 (1.23–4.52)		0.99 (0.58–1.69)	
**Employment status** ^**c**^								
Unemployed (reference)	1		1		1		1	
Paid	1.72 (1.08–2.73)	0.006	1.12 (0.52–2.42)	0.809	0.78 (0.51–1.20)	0.503	1.27 (0.87–1.86)	0.251
Unpaid	0.91 (0.54–1.53)		1.26 (0.61–2.64)		0.95 (0.56–1.60)		1.34 (0.91–1.98)	
**Age**								
25–34 y (reference)	1		1		1		1	
35–44 y	1.19 (0.84–1.69)	0.427	0.93 (0.49–1.76)	0.650	3.24 (1.53–6.85)	<0.001	0.62 (0.39–0.99)	<0.001
45–54 y	1.31 (0.80–2.16)		1.08 (0.58–2.01)		7.03 (3.73–13.28)		0.37 (0.22–0.63)	
55–64 y	0.87 (0.47–1.59)		0.64 (0.30–1.35)		12.98 (5.29–31.86)		0.44 (0.22–0.88)	

*Abbreviations*: FSM Federated States of Micronesia, BP Blood pressure, OR Odds ratio, CI confidence interval, y year, NA not applicable.

^a^Using the 2002 STEPS Pohnpei, FSM data set; all estimates are sex-age standardized to the FSM 2000 Pohnpei Census; P values based on the Rao-Scott adjustment to the χ^2^.

^b^Daily tobacco use includes daily use of cigarettes, cigars, pipes, or smokeless tobacco.

^c^Unpaid category includes student, homemaker, volunteer, or retired.

^d^Physically inactive includes <30 minutes/day of moderate activity on five or more days/week.

^e^High BP defined as BP ≥ 140/90 mmHG, or self-reports of BP medication use or hypertension diagnosis.

^f^Central obesity defined as waist circumference: Male > 102 cm; Female > 88 cm).

We examined age-stratified results (Table 5). Compared with other age groups, participants aged <35 years reported more varied patterns between socioeconomic position and CVD risk factors. For example, those in this group with a postsecondary educational level were less likely to report daily tobacco use (OR: 0.29, CI: 0.12–0.76, p = 0.024) than those with a primary educational level; those reporting high income (>$10,000) had over four times the odds of high BP (OR: 4.68, CI: 2.18–10.06, p < 0.001) than those with low income (<$5000). Compared with women in the same age ranges, men aged 25–34 years had nearly three times the odds (OR: 2.82, CI: 1.47–5.40, p < 0.001) and those aged 35–44 years had >4.5 times the odds (OR: 4.74, CI: 3.13–7.17, p < 0.001) of reporting daily tobacco use.

**Table 5:** **Age-stratified logistic regression models on association between socioeconomic demographic characteristics and cardiovascular risk factors, Pohnpei, FSM, 2002** ^**a**^

Age group	Predictor (measure)	Daily tobacco use ^b^		Physically inactive ^c^		High BP ^d^	
		OR (95% CI)	p	OR (95% CI)	p	OR (95% CI)	p
25–34 y	**Education (highest level completed)**						
	Primary (<9 y) (reference)	1		1		1	
	Secondary (9–12 y)	0.56 (0.30–1.01)	0.024	0.68 (0.42–1.01)	0.001	0.37 (0.16–0.87)	0.51
	Post-secondary (≥13 y)	0.29 (0.12–0.76)		0.29 (0.15–0.57)		0.62 (0.30–1.30)	
	**Income (estimated annual household)**						
	Low (<$5,000) (reference)	1		1		1	
	Middle ($5,000–$10,000)	0.66 (0.26–165)	0.272	1.09 (0.40–2.97)	0.629	1.61 (0.60–4.29	<0.001
	High (>$10,000)	0.43 (0.13–1.48)		2.32 (0.45–11.99)		4.68 (2.18–10.06)	
	**Employment status** ^**e**^						
	Unemployed (reference)	1		1		1	
	Paid	1.79 (0.91–3.24)	0.046	2.10 (0.98–4.48)	0.131	0.98 (0.51–1.90)	0.804
	Unpaid	0.81 (0.43–1.55)		1.28 (0.54–3.05)		1.43 (0.42–4.82)	
	**Sex**						
	Female (reference)	1		1		1	
	Male	2.82 (1.47–5.40)	0.001	0.23 (0.12–0.42)	<0.001	3.77 (1.83–7.79)	<0.001
35–44 y	**Education (highest level completed)**						
	Primary (<9 y) (reference)	1		1		1	
	Secondary (9–12 y)	0.86 (0.56–1.32)	0.161	1.00 (0.53–1.89)	0.606	0.98 (0.54–1.79)	0.854
	Post-secondary (≥13 y)	0.51 (0.24–1.05)		0.66 (0.28–1.55)		0.76 (0.28–2.07)	
	**Income (estimated annual household)**						
	Low (<$5,000) (reference)	1		1		1	
	Middle ($5,000–$10,000)	1.00 (0.46–2.15)	0.563	1.24 (0.65–2.36)	0.887	0.71 (0.30–1.70)	0.188
	High (>$10,000)	0.54 (0.16–1.56)		1.47 (0.34–6.39)		2.07 (0.81–5.28)	
	**Employment status** ^**e**^						
	Unemployed (reference)	1		1		1	
	Paid	0.82 (0.45–1.51)	0.776	1.04 (0.48–2.24)	0.114	1.19 (0.75–1.90)	0.478
	Unpaid	0.87 (0.44–1.70)		2.19 (0.85–5.66)		0.74 (0.33–1.66)	
	**Sex**						
	Female (reference)	1		1		1	
	Male	4.74 (3.13–7.17)	<0.001	0.17 (0.17–0.65)	0.001	1.62 (0.91–2.91))	0.089
45–54 y	**Education (highest level completed)**						
	Primary (<9 y) (reference)	1		1		1	
	Secondary (9–12 y)	0.76 (0.43–1.33)	0.592	1.51 (0.68–1.23)	0.445	1.01 (0.61–1.67)	0.769
	Post-secondary (≥13 y)	0.91 (0.38–2.19)		1.65 (0.39–6.95)		1.23 (0.65–2.30)	
	**Income (estimated annual household)**						
	Low (<$5,000) (reference)	1		1		1	
	Middle ($5,000–$10,000)	0.83 (0.34–2.02)	0.115	1.17 (0.42–3.23)	0.518	0.88 (0.51–1.50)	0.604
	High (>$10,000)	0.23 (0.07–0.80)		0.96 (0.23–3.88)		1.38 (0.77–2.46)	
	**Employment status** ^**e**^						
	Unemployed (NA) (reference)	1		1		1	
	Paid (NA)	1.70 (0.93–3.11)	0.074	0.91 (0.44–1.90)	0.923	0.70 (0.39–1.27)	0.473
	Unpaid (NA)	2.33 (1.02–5.33)		1.17 (0.41–3.33)		0.71 (0.33–1.51)	
	**Sex**						
	Female (reference)	1		1		1	
	Male	2.54 (1.48–4.35)	<0.001	0.47(0.21–1.04)	0.051	1.65 (1.01–2.65)	0.031
55–64 y	**Education (highest level completed)**						
	Primary (<9 y) (reference)	1		1		1	
	Secondary (9–12 y)	0.68 (0.18–2.68)	0.857	1.00 (0.20–4.93)	0.884	1.02 (0.43–2.42)	0.767
	Post-secondary (≥13 y)	0.85 (0.18–4.14)		0.76 (0.19–3.02)		0.61 (0.14–2.61)	
	**Income (estimated annual household)**						
	Low (<$5,000) (reference)	1		1		1	
	Middle ($5,000–$10,000)	0.54 (0.12–2.33)	0.183	0.54 (0.20–1.48)	0.143	1.49 (0.76–2.91)	0.441
	High (>$10,000)	1.98 (0.63–6.21)		1.00 (0.35–2.89)		1.89 (0.64–5.56)	
	**Employment status** ^**e**^						
	Unemployed (reference)	1		1		1	
	Paid	1.53 (0.49–4.78)	0.723	0.50 (0.14–1.82)	0.088	0.73 (0.31–1.77)	0.626
	Unpaid	1.15 (0.27–4.92)		1.27 (0.38–4.26)		0.72 (0.36–1.45)	
	**Sex**						
	Female (reference)	1		1		1	
	Male	0.98 (0.37–2.62)	0.968	1.00 (0.42–2.37)	0.994	1.70 (0.79–3.69)	0.159

*Abbreviations*: *FSM* Federated States of Micronesia, *BP* Blood pressure, *OR* Odds ratio, *CI* Confidence interval, *y* year, *NA* Not applicable.

^a^Using the 2002 STEPS Pohnpei, FSM data set; all estimates are sex-age standardized to the FSM 2000 Pohnpei Census; P values based on the Rao-Scott adjustment to the χ^2^.

^b^Daily tobacco use includes daily use of cigarettes, cigars, pipes, or smokeless tobacco.

^c^Physically inactive includes <30 minutes/day of moderate activity on five or more days/week.

^d^High BP defined as BP ≥ 140/90 mmHG, or self-reports of BP medication use or hypertension diagnosis.

^e^Unpaid category includes student, homemaker, volunteer, or retired.

## Discussion

Our analysis revealed that socioeconomic position and demographic characteristics were associated with selected CVD risk factors with variations by risk factor, sex and age characteristics, and in the direction of the association (i.e., direct or inverse).

### Related studies

Worldwide, as of 2006, smoking prevalence was higher for men (40%) than for women (nearly 9%), and men accounted for 80% of all smokers [30]. Our findings were consistent with a recent review of data from 48 LMICs participating in the World Health Survey, which revealed higher smoking rates among the less educated, decreasing proportions of smoking among persons in older age groups, and inverse associations between wealth and smoking within LMICs [31]. In contrast, our analysis found that, when controlling for other factors, women reporting paid employment and men reporting unpaid employment had twice the odds of daily tobacco use than those reporting unemployment.

Although other studies have assessed a variety of physical activity domains, in general our findings of higher likelihood of low physical activity for women, increasing age group, and those reporting primary education are consistent with other studies in LMICs. For example, an assessment of cross-national data from Estonia, Latvia, and Lithuania revealed that lower educational level is a strong and consistent predictor of leisure time inactivity across gender [32]. In most countries, men are more active than women [33, 34], and increasing age is associated with lower physical activity prevalence, especially in men [34].

In our study’s general population a higher income and paid employment status were associated with higher odds of high BP and central obesity, respectively. We also observed differences across gender. For example, men had twice the odds of high BP than women and women with incomes > $10,000 had more than twice the odds of high BP than those reporting incomes < $5,000 though not for men. We also found that women had twice the odds of central obesity than men. For men, paid employment was associated with three times the odds of central obesity. Some other studies in LMICs have reported different results for these associations. For example, in rural Vietnamese adults, aged 25–64 years, hypertension was inversely associated with education level [35]. Another study among urban Latin American adults aged *≥*18 years found inverse associations for income and education with hypertension [36]. Additionally, in a landmark review (1989) on the relationship between socioeconomic position and obesity, researchers reported a strong inverse association for women in developed countries, with a higher likelihood of obesity among women in lower socioeconomic groups [37]. In 2007, McLaren [38] updated this review, reporting a gradual reversal of this social gradient (for both men and women), with an increasing proportion of positive associations and a decreasing proportion of negative associations when shifting from high-income countries to medium- and low- income countries.

While evidence is limited, the varied patterning among socioeconomic position and CVD risk factors in our study may suggest a gradual shift in the epidemiologic transition within Pohnpei. For many high-income countries, thru the progression of socioeconomic development, researchers have documented an epidemiological transition; from a direct to an inverse association between socioeconomic position and CVD risk factors [15]. Rising rates of mean BP has been identified as an early indicator of this transition [6]. Comparisons with previous research, although limited, suggest a rise in mean BP among adults living in Pohnpei. For example, seminal cross-sectional studies in 1947 and 1953 observed low BP among adults in Pohnpei and, in 1983, researchers reported significantly increased diastolic BP among urban males [39]. While our findings suggest a continued increase in mean BP among adults living in Pohnpei, particularly for those reporting higher income.

Researchers suggest that the epidemiologic transition occurs because wealthier and more educated persons tend to be early adopters of high-risk behaviors that contribute to CVD [40, 41]. As health-related consequences are realized, those in higher socioeconomic position embrace lifestyle change and other prevention measures, subsequently lowering their CVD risk. In contrast, the uneducated poor may experience later peaks in higher risk lifestyle behaviors—such as less physical activity, high-fat diets, or increased psychosocial stress—that lead to increased CVD after declines are seen among the wealthier [40]. Unlike high-income countries, the challenge for LMIC countries is that this transition may occur more rapidly with the changes in risk factors and CVD prevalence outpacing the development of policy, environmental and system changes needed to effectively target CVD reduction [6].

### Implications for CVD risk factor prevention

In 2011, a political declaration from the United Nations high-level meeting on chronic noncommunicable diseases recommended stronger country-level surveillance and monitoring of chronic diseases and associated risk factors, including socioeconomic determinants, to appropriately target public health policy and programmatic needs [42]. Although repeated surveys will provide trend data for CVD risk factors, our study results demonstrate how population health surveillance data can provide context-specific strategies to address CVD risk factors in that population, in this case that of Pohnpei. Over the last several years, the FSM Department of Health and Social Affairs has been building capacity to prevent and control chronic disease [43] and developing policy and environmental strategies to promote physical activity, consumption of local fruit and vegetables, and tobacco-free living, in partnership with governmental and community networks [43–45]. Possible interventions to address CVD risk factors within Pohnpei include:

-  Work-site wellness programs that provide access to and support for tobacco cessation and obesity prevention among adults.
-  Increased policy and promotional efforts to create smoke-free environments, and media campaigns and other governmental actions to reduce the social acceptability of tobacco use.
-  Environmental policies and programs aimed toward men in workplace settings and within cultural support networks, which encourage increased physical activity and healthy food choices to lower central obesity.
-  Enhanced BP monitoring and treatment at worksites, in health systems, and through community linkages (e.g., faith-based and men’s and women’s groups), targeting adults aged ≥35 years.
-  Collaborations with local communities to improve culturally and linguistically appropriate health literacy materials to raise awareness of CVD risk factors (i.e. tobacco use, low physical activity, high BP, and central obesity) and actions necessary to lower individual risk.

### Further research needs

While the 2002 Pohnpei STEPS population health survey establishes baseline data, monitoring time trends can provide a consistent, up-to-date, and standardized database to support population health research, policy, and program development. While our analysis revealed several significant predictors for CVD risk factors, the strengths of the associations were small, suggesting that other determinants, not examined in our study, may play important roles in the health of the population. Further research to assess the behavioral, biologic, and environmental impact of socioeconomic position on CVD risk-factor clustering may also help increase understanding and inform population health efforts to reduce the burden of CVD disease [15, 46–48] in the people of Pohnpei.

### Strengths and limitations

The strengths of this study include engagement of the FSM Department of Health and Social Affairs leadership, a representative sample, and objective anthropometric measures. We also acknowledge the study limitations. Inherent methodological biases in our study model limit the generalizability of findings beyond Pohnpei and other subpopulations in FSM. For example, because the STEPS dataset did not provide probability variables for sample selection, we assigned standardized age-sex rates, which may not have been representative of the 2002 adult Pohnpeian population.

Additionally, we used a cross-sectional sample for this study, which does not allow examination of causal relationships. The study also includes use of self-reported behavioral risk-factor measures that are subject to participant recall, social desirability, and response bias. The limited data collection period, within the primary study, may have also introduced seasonal variation in responses. These potential biases could contribute to under- or over-reporting of risk [49].

## Conclusion

In LMICs, the burden of CVD morbidity and mortality has been increasing at more compressed rates than those experienced by high-income countries in previous decades. Country-level population health surveillance is critical for understanding the epidemiology of CVD risk factors. Our results indicated that, in Pohnpei, socioeconomic position and demographic characteristics were associated with selected CVD risk factors with variations by risk factor, sex-age characteristics, and in the direction of the association. The 2002 Pohnpei dataset provided country-level baseline information; to determine trends, further population health surveillance and monitoring is needed. Having trend data might help decision makers tailor policy, program interventions, and fiscal resource needs for CVD prevention.

## Electronic supplementary material

Additional file 1: **Assessment of the 2002 Pohnpei STEPS dataset: Criterion, definition, and application to the secondary analysis.** (DOCX 41 KB)
