# Supplementary material for: Socioeconomic and demographic predictors of selected cardiovascular risk factors among adults living in Pohnpei, Federated States of Micronesia
Source: BMC Public Health. 2014 Aug 31;14:895. doi: 10.1186/1471-2458-14-895 (PMC4158138; doi:10.1186/1471-2458-14-895)
Supplement: Supplementary file 1 — Additional file 1: Assessment of the 2002 Pohnpei STEPS dataset: Criterion, definition, and application to the secondary analysis. (DOCX 41 KB) [file 12889_2013_7004_MOESM1_ESM.docx]

**Additional file 1** Assessment of the 2002 Pohnpei STEPS dataset: Criterion, definition, and application to the secondary analysis

| **Assessment criterion ^a^** | **Definition** | **Application to the secondary analysis** |
| --- | --- | --- |
| **Overall design** | Availability of:  1) Metadata ^b^ | Public website STEPS metadata (www.who.int/chp/steps/en/) includes:  1) Design and protocol(s); 2) user manuals; 3) codebook; 4) instruments; 5) other resources (i.e., training modules, country-level summary reports, and templates) |
|  | 2) Microdata ^c^ | - Public access to 2002 FSM (Pohnpei) STEPS summary report. - Data use agreement and IRB approval need for microdata release - Data file readable using ASCII format. |
| **Methodology** | Review of:  1) Sample design | - Multistage probabilistic cluster sample design using 2000 FSM Pohnpei Census enumeration districts; Representative household sample (one individual per household) of adults (25–64y) |
|  | 2) Representation | - Sample size calculations to detect prevalence rates of approximately 20% (CI±2%) and differences between age/sex groups with CI±10% for noncommunicable disease risk factors suggested a total sample of 1650 participants. A total of 2100 participants were targeted for the survey and physical measures with approximately 30% selected for biochemical tests. - Primary dataset N=1638 (78% response rate); technical variables available to allow for age/sex standardization; sample design detail not available to adjust for probability of selection |
| **Instrument** | Review of:   1. Variable definitions | Variable definitions (i.e., conceptual and operational) were relevant to research hypothesis. One proxy variable defined (i.e., health access) using health screening questions available in data set. |
|  | 1. Contextual issues within STEPS survey | Contextual issues that may impact responses (leading to nonresponse or missing data) include:  1) cultural relevance and comprehension of questions, 2) timing of data collection 3) self-reported responses, and 4) participant burden. However, these issues may be limited as the survey was conducted: 1) by trained staff, 2) face-to-face in the participant’s home or local clinic, 3) in English or local language, and 4) day & evening. |
| **Data file** | Review of   1. Data file structure and mapped instrument | Microdata file mirrors data collection phases. Initial descriptive tabulation of data found inconsistencies in mapped instrument and data coding/entry. For example, education categories did not consistently match with reported years of education, thus re-categorization of education was necessary. |
|  | 1. Missing data | Generally, selected variables had <10% missing data. However, an “unknown” income category was created to account for excessive missing values (N = 405, 24.7%). |

Abbreviations: FSM Federated States of Micronesia; IRB Institutional Review Board; y years; CI confidence interval; N sample size

^a^ Evaluation criteria sources [22-24]

^b^ Metadata is defined as structured information that describes, locates, and helps retrieve data resources

^c^ Microdata is defined as original survey sample (i.e., raw data)
